# Effect of boiler oversizing on efficiency: a dynamic simulation study

**DOI:** 10.1177/0143624420927352

**Published:** 2020-05-22

**Authors:** George Bennett, Cliff Elwell

**Affiliations:** 4919 University College London, London, UK

**Keywords:** Building energy simulation, buildings energy performance, domestic buildings, space heating, boiler

## Abstract

Gas boilers dominate domestic heating in the UK, and significant efficiency improvements have been associated with condensing boilers. However, the potential remains for further efficiency improvement by refining the control, system specification and installation in real dwellings. Dynamic building simulation modelling, including detailed heating system componentry, enables a deeper analysis of boiler underperformance. This paper explores the link between the space heat oversizing of boilers and on/off cycling using dynamic simulation, and their subsequent effect on boiler efficiency and internal temperatures. At plant size ratio (PSR) 8.5 daily cycles numbered over 50, similar to median levels seen in real homes. Simulations show that typical oversizing (PSR >3) significantly increases cycling behaviour and brings an efficiency penalty of 6–9%. There is a clear link between raising PSR, increased cycling and an associated decreased efficiency; however, in the UK, boilers are regularly oversized with respect to space heating, especially combination boilers to cover peak hot water demand. Current legislation and labelling (ErP and SAP) overlook PSR as a determinant of system efficiency, failing to incentivise appropriate sizing. Reducing boiler oversizing through addressing installation practices and certification has the potential to significantly improve efficiency at low cost, decreasing associated carbon emissions.

**Practical application:** This research provides the basis for a practical and cost effective means of assessing the potential for underperformance of boiler heating systems at the point of installation or refurbishment. By assessing the oversizing of the boiler with respect to space heating, unnecessary cycling and the associated efficiency penalty can be avoided. Plant size ratio, as an indicator of cycling potential, can be implemented in energy performance certificates (EPCs), through the standard assessment procedure (SAP), using existing data. The potential for real carbon savings in the existing boiler stock is considerable, and the findings have wider implications for next generation heating systems.

## Introduction

The UK residential heating landscape is dominated by one technology: the gas boiler. In 2007, boilers accounted for 86% of the heating systems of England^1^ totalling over 20 million appliances, and boilers are being installed (new and replacement) at a rate of 1.2 million per year.^2^ Small improvements in the efficiency of gas boiler heating can have a major impact on national emissions; yet, a persistent performance gap between predicted and actual boiler energy demand remains,^3–5^ of the order of a 10% efficiency drop. Closing this performance gap has the potential to significantly and rapidly decrease carbon emissions and energy use.

Boiler systems in occupied dwellings have been shown to exhibit cycling behaviour that is associated with reduced overall efficiency.^6^ The tendency for combi boilers to be oversized with respect to central heating was noted from the large typical boiler thermal output sizes compared to the heat demand of the stock. This oversizing is the probable cause of observed cycling: high numbers of short heating cycles were observed.^6^ Although those observations are consistent with the circumstances that would lead to efficiency losses,^7^ the link between system efficiency and cycling behaviour has been observed but not fully explored in previous studies.^3^

This paper aims to address this research gap by simulating boiler system performance as the plant size ratio varies, and therefore relative oversizing of the heating system varies, in addition to exploring ways to avoid or mitigate the negative consequences of oversizing.

## Literature review

The advent of boilers with variable rate/modulating power output levels enabled the combination of direct hot water and space heating in the same appliance. On demand, hot water requires rapid control of the heat input in order to deliver consistent hot water temperature despite a variable flow rate and cold feed temperature. Combining the two functions saves space and reduces installation complexity, by eliminating the need for storage. Peak hot water demand is proportional to the maximum flowrate expected, which is often considered proportional to the number of occupants or bathrooms,^1^,^2^ whereas the space heating demand is derived from the heat loss of the building. In practice, peak hot water demand is mostly significantly greater than the space heating demand, so the design, sizing and selection of combi appliances are based on domestic hot water (DHW) capacity. For example, a boiler installed in a dwelling with two bathrooms would be required to deliver up to 13 L/min of DHW (at a temperature increase of 40 K), necessitating 30–36 kW of DHW capacity.^8,9^ Studies of real energy demand and survey data^10^ estimate the mean actual space heat load of a UK dwelling to be around 6–8 kW, with an internal-external temperature difference of 23°C,^3^ corresponding to a cold winter day. A combination boiler should be capable of meeting these significantly mismatched space and water heating outputs.

The (mis)matching of boiler output to heat demand is commonly quantified by means of the plant size ratio (PSR), a succinct term to refer to the ratio of maximum heater thermal power output (${\dot{Q}}_{H})$ to the building design steady state heat loss (${\dot{Q}}_{B}$)

(1) $$\mathrm{PSR}=\frac{{\dot{Q}}_{H}}{{\dot{Q}}_{B}}$$

Space heating design load (${\dot{Q}}_{B})$ is calculated based on the steady state building heat loss for a chosen temperature difference across the building fabric (with heat transfer coefficient, $U\;(W/m^{2}K)$ and total surface area, $A\;{(m}^{2})$) accounting for ventilation losses (with coefficient, $C_{v}(\frac{W}{K})$) . A design day with 21°C internal and –2°C external temperature is typical, although a regional calculation method is recommended in BS EN ISO 15927-5 based on historical coldest months, which could lead to design external temperatures between approx. 0 and –6°C (ashrae-meteo.info).

The below equation shows the building space heat loss for plant size calculation^11^

(2) $${\dot{Q}}_{B}=\sum \left(\mathrm{UA}\Delta T\right)+C_{v}\Delta T$$

If the system is expected to provide constant heating, a boiler thermal output may be selected directly on the basis of the design day heat demand. However, heating schedules are often operated intermittently due to occupancy, comfort requirements or tradition.^12^ Accordingly, a heating system multiplication factor is used to account for the cooling that will occur outside of the heat schedule and the extra thermal power required to return the heated space to the required temperature within a reasonable time.^13^

CIBSE offers a simple set of discrete multiplication factors to identify the required design heat load, separating buildings into fast or slow thermal response, based on construction type, inferred thermal mass and thermal time constant.^11^ The factors replicated in Table 1 show that for buildings with 12 or more hours of continuous heating, no adjustment to the plant size is deemed necessary regardless of building thermal response. When the heating schedule is shorter, notable increases in plant size are recommended for fast thermally responding houses, up to a practical maximum of 2.8.

**Table 1.** Plant size multiplication factors according to building thermal response a ratio of cyclic response to thermal transmittance.^11^

Daily hours of heating ON time $t_{0}$	Multiplication factor acc. building thermal response	
	Slow	Fast
12	1.0	1.0
6	1.1	2.0
4	1.2	2.8

**Figure 1.**
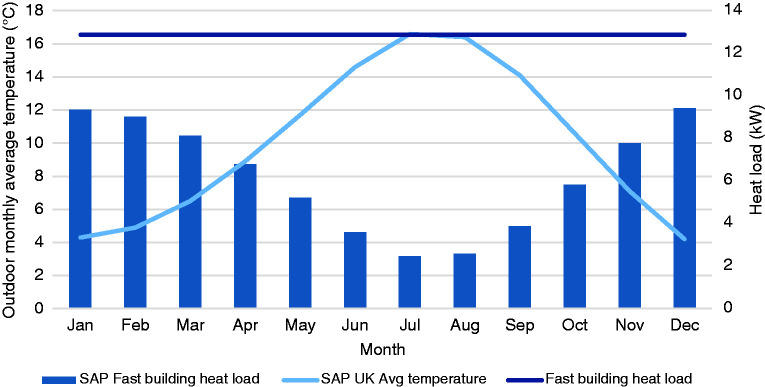
Design day and temperature dependent building heat load with CIBSE factors for intermittent operation, ‘fast’ low thermal lag building shown. SAP: standard assessment procedure.

In practice, the required heater power requirement (with a fixed multiplication factor relating to the building fabric) varies with time as the building heat loss changes with outdoor temperature (in contrast to the PSR which is fixed and dependent on specified boiler thermal output and theoretical peak building steady state heat loss). Since the design day heat load is chosen to account for the coldest expected days, the actual heat load for the majority of days in any winter is expected to be significantly lower, as shown by Figure 1. This issue is exacerbated in milder winters, with a correspondingly lower building heat load and larger mismatch, and in the shoulder seasons of each year.

Modulation of the boiler heat output aims to match this heat demand and supply. Current pneumatically controlled premix gas valve technology is limited to a modulation ratio of approximately 1:10 in newer boilers,^14^ with 1:6 being more common. A practical result of this limited modulation range is that a boiler with maximum thermal output of 36 kW can normally modulate to a minimum of 6 kW.^15^ This suggests that it is likely that combi boiler heating systems have to cycle on and off to match the space heat demand, with implications for the in situ efficiency.

Although the efficiency of gas boilers is relatively robust regarding part load operation, it is not independent thereof, with testing for product energy labelling reflecting that fact, with measurements at full and 30% load.^13^ Boiler efficiency, as a percentage of useful heat produced relative to energy consumed, is subject to the operating conditions of the boiler at that time. Important factors include the gas/air ratio, flue gas temperature (itself a function of heating water temperature and heat exchanger effectiveness) and heat power modulation level. Furthermore, on/off cycling is known to be a major influencer of efficiency, but is less well understood in practice. Trials, such as the BRE condensing boiler assessment,^4^ have explored the real efficiency of boilers in operation and have led to assumptions around efficiency adjustments that find their way into the calculation methods, such as those for the standard assessment procedure (SAP) used in energy performance certificate (EPC) generation.

The influence of the thermal output range of a boiler on efficiency in practice is covered from a theoretical perspective in handbooks and guidelines for professionals in the field, e.g. the Buderus Handbuch für Heiztechnik.^16^ These texts describe that standby losses may be greater for a larger boiler due to increased surface area of the boiler itself; however, this maintains focus on steady state conditions and not on real dynamic operation. Performance factors such as start-up/shutdown sequences, standby and running losses are inherently dynamic. Start/stop losses, associated with the switching on and off of boiler operation during a scheduled heating period, become more significant with short cycles. Orr et al. reported an exponential relationship^3^ rising from 1.5% for 3 min boiler runtime to 11.8% for 10 s runtimes. The same study reported widespread underperformance of boiler systems, oversizing of combination boilers and a correlation between low monthly load factor and decreased efficiency. Confirmation of the prevalence of oversizing and short operation cycles has come from analysis of high frequency boiler diagnostic data,^6^ in this large dataset of 209 dwellings 20% of boilers averaged less than 2.5 min per firing. A limited case study found that 25% of all firings for combi boilers were less than 2 min.^17^

Current efficiency testing of boilers for space heating is conducted at steady state and the representative efficiency, which is placed on the energy label, can be arithmetically derived from two steady state load conditions with a weighted average. Tests are conducted at maximum and 30% power modulation levels using controlled flow and return temperatures; the results are combined in a weighted average (30:70 maximum to lower modulation measurement). In contrast, the efficiency measurement for the DHW is based on a hot water demand (tapping) schedule (EN15502), which simulates typical daily hot water demand schedules incorporating various tappings of different flowrates and temperatures in the time domain and ensuring the dynamic response of the boiler is captured.

To simplify the functional efficiency of a boiler and its system into a representative value is a challenge, made more complex when such a value may be utilised for multiple aims, e.g. product labelling, consumer comparison and standardised building energy assessment (SAP). SAP uses the singular figure of the Seasonal Efficiency of Boilers in the UK (SEDBUK) rating (if available in the Product Characteristic DataBase (PCDB)^18^) as the starting point of its procedure to calculate gas demand from the building heat load. Adjustments are made to decrease the assumed efficiency of all boilers due to previously observed underperformance.^4^ Positive adjustments are made according to other factors deemed beneficial to system performance, such as low temperature emitters and modern controls. However, the methodology inherits the assumption in the original SEDBUK efficiency that the boiler efficiency can be derived from steady state measurements. Inclusion of real-world dynamic behaviour in the efficiency estimation of boilers can support improved energy labelling, installation quality and reduced energy bills for consumers.

Recent simulations of the dynamic performance of heating systems^19^ have shown that standardised, steady state, methods to estimate energy use do not accurately capture the heat demand, supply and internal temperatures. However, the relationship between the dynamic behaviour of boiler-based heating systems and their efficiency has not been widely studied. Dynamic simulation and field studies of heating system performance can be used to investigate these relationships in detail, including their impact on efficiency. Furthermore, potential mitigating strategies, for existing and future boiler systems, can be explored, which may also pave the way for the next generation and emerging heating systems in the UK, such as heat pumps, and the transition to low carbon heating. This paper seeks to bridge the gap between observed boiler underperformance, dynamic behaviour and boiler operating/installation parameters. From within a simulation environment, the suspected important performance driver of PSR was varied systematically to induce the cycling behaviour previously seen in the field and the impact on boiler efficiency quantified. The magnitude of underperformance attributable to oversizing was assessed, possible mitigation strategies discussed and methods for integrating the new knowledge into installer practice and policy tools explored.

## Methods

This paper systematically addresses, in a simulation environment, the efficiency impact of boiler sizing, using the example of a common UK house type and explores some means of mitigating the challenges of over and under sizing boilers. Simulated heating operation addresses the widely reported intermittent heating schedule in UK residential dwellings^20^ and the evidence of general oversizing of boilers,^3^ which is associated with high levels of cycling.^6^

A boiler cycle is defined as an operational period which contains both one central heating ON (>0% modulation) period and one OFF period (0% modulation), where the boiler operation is not interrupted by user intervention in the form of hot water demand or similar. Since the simulation covered only space heating operation, all cycles are categorised as central heating (CH) cycles and are therefore determined by the interaction between the characteristics of the space heating system and the building heat demand. The simulations performed aim to strengthen the depth of understanding around dynamic behaviour of domestic heating systems and point the way to improving real-world performance. Below, firstly, the simulation environment is discussed, followed by the simulation parameters for both the house and the heating system.

### BTSL Simulation environment: TRNSYS and Simulink

**Figure 2.**
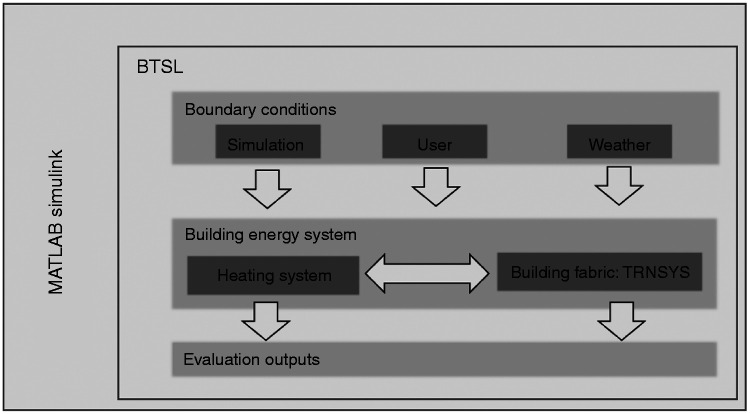
Schematic representation of hierarchy in BTSL simulation environment. BTSL: Building Technology Simulation Library.

The BTSL (Building Technology Simulation Library) model is a fully dynamic engineering model with a library of simulation blocks such as archetypes of buildings, heating system components and users, which can be linked within the MATLAB Simulink environment; the interaction of these elements is shown in Figure 2. BTSL operates as a co-simulation between TRNSYS building model and MATLAB-based heating and user simulation. BTSL allows for modular creation of a building model, whereby the heating system and building characteristics, user behaviour and weather can be chosen. The aim of such co-simulations is to allow developers to extend the scope of simulations by adding simulation blocks of different types and depth to the central building model. This type of hybrid simulation environment is also possible with the popular EnergyPlus building simulation software^21^ using the Building Control Virtual Test Bed (BCVTB).

BTSL is a proprietary heating system emulation tool for product development at Bosch Thermotechnology, developed from their previously validated *LabHouse* tool.^22,23^ It has been developed and expanded to include a wide range of heating ventilation air conditioning (HVAC) components such as radiators, thermostatic valves, heat exchangers, cooling coils, fans and pumps: the depth of detail at which a user can specify the heating system is a key advantage to BTSL over publicly available tools. In addition, the transient behaviour of the heating appliance is modelled through time response parametrisation, control feedback loops and the associated control algorithms. This type of proprietary modular concept is used in industry to simulate heating systems under a number of installation environments and verify performance and control strategies. An analogous modular construction of simulation in the MATLAB environment with a TRNSYS Building model has been suggested,^24^ which served the purpose of evaluating the possible intervention options, building and HVAC system, available in a building upgrade situation.

BTSL is designed to support the development of heating systems and their controls and thus has a high level of flexibility with regard to the heating system library block in Simulink, and uses an existing building model, TRNSYS,^25^ to simulate the building fabric. The TRNSYS building model, known as “Type 56”, is a modular transient system simulation program which meets the general technical requirements of the European Directive on the Energy Performance of Buildings, making TRNSYS a potential candidate for compliance with the directive’s implementations in various EU countries.

BTSL is utilised in this paper to explore how a fully dynamic national calculation method, such as the UK’s Standard Assessment Procedure, would reveal interactions between boiler, PSR and controls. The model used here builds on previous research into the dynamic performance of heating systems; utilising the same building model and many of the same heating system components^19^ the BTSL model was able to reproduce SAP results, with some adaptation of simulation blocks.

The modelling strategy built on using dynamic inputs (weather, solar gain, setpoint temperatures) in dynamic representations of the physical elements of the building/heating system. Most aspects were implemented using standard components of the library, such as the dynamic weather, building and heating system models. Using this approach, the model can draw directly on the same control algorithms implemented in Bosch heating appliances and the thermal response characteristics of Bosch boilers.

The simulated weather was taken from the US Department of Energy database for the UK town of Finningley (USDoE, 2013, US Department of Energy Weather Data files, https://energyplus.net/weather-location/europe_wmo_region_6/GBR//GBR_Finningley.033600_IWEC) providing hourly dynamic inputs of solar radiation, wind speed, ground and air temperature. Certain elements of the model were kept constant with time, although the use of dynamic time series representations was possible. Aspects kept uniform with time included gains from electrical appliance use, cooking and metabolism. Holding such factors constant enables closer comparison with the outputs of a SAP model of the building, but retains the key factor of modelling the dynamic heating system performance with varying heat lead.

### House and heating system parameters

The chosen test case is a detached east-facing two-storey house with an above average standard of efficiency (C80 rated EPC) and equipped with a typical gas combi-boiler heating system. The house model is the same as used in previous research^19^ within the BTSL simulation environment.^22^ A summary of the building properties is listed in Table 2.

**Table 2.** Selected building and heating system simulation parameters.

Parameter	Value	Unit
SAP parameters		
HLP (heat Loss Parameter)	1.3652	W/m^2^K
TMP (thermal mass parameter)	283	kJ/m^2^K
TFA (total floor area)	100	m^2^
Living area (Zone 1)	30	m^2^
Window area	23	m^2^
Window orientation	East	–
Main heat source	Gas Combi Boiler (Condensing nominal 90% efficiency, size varied in simulations) Modulation range 1:5	
Setpoint room temperature	21	°C
Heating schedule weekdays	07:00–09:00 16:00–23:00	
Heating schedule weekends	07:00–23:00	
Heating system emitter type	Radiators (sized according to 80/60 flow/return temperatures)	–
Heating system control	Programmer, Room Thermostat and Thermostatic Radiator Valves (TRVs)	–

The building, as modelling in TRNSYS within BTSL, was a simple four room layout (two per floor) with the front room on the ground floor designated as the main living space (30% of total floor area as in Table 2). The construction comprises cavity-filled walls (U = 0.19W/m^2^K), dual-pitched warm roof (U = 0.13W/m^2^K), solid floor (U = 0.18W/m^2^K) and double-glazed windows (U = 0.12W/m^2^K), the overall building thermal characteristics are in Table 2. The combi boiler was taken from the BTSL library, which uses a physically representative model of the thermal characteristics, derived from the lab testing of a major boiler manufacturer. The boiler model also includes all necessary ancillary components, such as fans, pumps and control systems. Importantly for the analysis, this includes real-world boiler control algorithms such as start-up sequences, ramp rates, an anti-cycling control. This detailed model allows for realistic accounting for the electrical energy consumed during boiler operation, which is then used in the efficiency calculation together with the gas consumption. The control algorithms are also representative of commercially available appliances in the UK.

Applying the UK standard SAP analysis to the simulated dwelling, the design day heat load is 3.3 kW, used for PSR calculations in Table 3, which contains the range of PSRs and control parameters simulated in the BTSL environment with the house described in Table 2. The range of PSR was informed by the general observations of installed combi boilers in the UK^3,6^; it ranges from highly oversized (PSR 8.5) down to theoretically undersized boilers of PSR 0.5.

**Table 3.** Summary of parameter space covered by simulations.

Parameter	Options/Range	Notes
Plant size ratio	8.53.02.01.00.5	Defined as:Ratio of boiler rated output/ building design day heat loss at an external temperature of –2°C, excluding free heat gains.
Heat up optimisation	ON or OFF	Control algorithm which activates the heating system before the scheduled time in order to achieve the desired internal temperature at that time, avoiding delayed heat up.

A PSR of 8.5 corresponds to a 28 kW boiler, a typical maximum thermal output of combi boilers (corresponding to ∼10 L/min DHW capacity).^6^ Smaller sizes (corresponding to PSR less than 8.5 in this simulated case) are not commonly included in the product offerings of major manufacturers,^26^ because they are not able to provide ‘on demand’ DHW at a high enough flow rate to meet expected customer requirement. The range of PSR used in this simulation does not include higher PSR than typically installed for a property of the type modelled, but does include significantly lower PSR than generally available, to explore its impact on operational efficiency (dictating the minimum operational power level of the boiler).

Reducing the size of a boiler will increase the time taken for a dwelling to reach thermostat set-point; therefore, ‘heat up optimisation’ was included in the simulation options to investigate a possible means of compensating for boiler size. ‘Heat up optimisation’, available in some modern controls, interprets the heating schedule not as the strict definition of heating system activity but as a literal interpretation of the desired internal temperature and adjusts the operation of the heating system appropriately. Heat up times are moved outside of the heating schedule, with the aim of delivering the desired room temperature at the required time. This will inevitably increase the duration of heating system operation, impacting gas consumption, but may reduce the need for oversizing, with consequent reductions in boiler cycling and potential improvements in efficiency.

## Results

**Figure 3.**
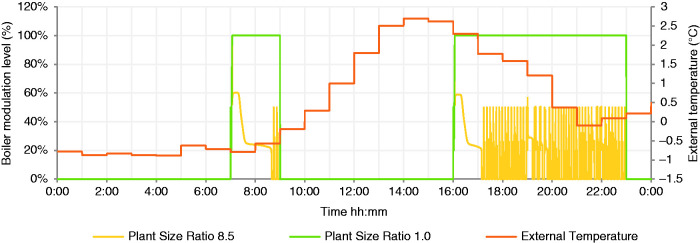
January day, PSR 8.5 and PSR 1 boiler modulation levels.

**Figure 4.**
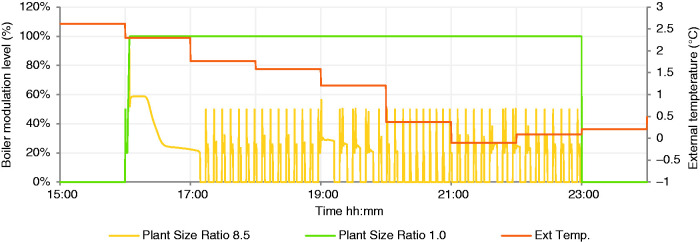
Detail of boiler modulation on January day, PSR 8.5 and PSR 1.

Simulations were carried out on the 10 parameter combinations listed in Table 3, for one full calendar year capturing the internal temperatures, heat demand, gas consumption and the dynamic response of the heating system as displayed in a representative January day in Figure 3. Although the simulation was conducted for a full year, this figure illustrates in a single day the impact of a high, 8.5 PSR, thermal output boiler, with finite modulation range. The boiler does not operate at full power after the first seconds of start-up and, after an initial heating up period, the boiler enters extended periods of cycling characterised by repetitive periods of boiler operation separated by boiler inactivity, on a timescale of minutes, seen more clearly in Figure 4. In contrast, the PSR 1 system operates at full power continuously throughout the morning and evening heating periods; the intermittent heating schedule is not suited to this low PSR: it is undersized according to industry norms (CIBSE).

## Cycling and efficiency

**Figure 5.**
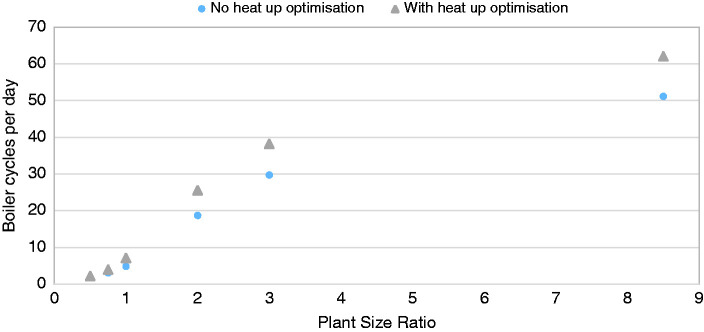
Cycles per day across PSR, with and without ‘heat up optimisation’.

By aggregating over the simulated year, the predicted cycling behaviour across the PSR range can be compared, as shown in Figure 5. It shows an increase in cycling behaviour as the PSR increases. Figure 5 also shows that the ‘heat up optimisation’ algorithm increases the number of observed cycles of boiler operation at a given PSR, which is explored below.

A heating system, which is continually and ideally matched to the changing thermal demand of the dwelling throughout the heating season, following the instantaneous heat demand, would be expected to average at most two cycles per day, coinciding with the start and end of each heating period, with no cessation of heating within the period. Such an appliance would need to be infinitely modulating within the range of building heat demand and able to react instantaneously. Figure 5 shows that only the smallest PSR of 0.5 results in continuous heating operation during the daily heating periods. Over 50 cycles per day were observed in the PSR 8.5 simulation, a good match to the median value of 53 from field data,^6^ where boiler sizes were in the range 25–31 kW.

**Figure 6.**
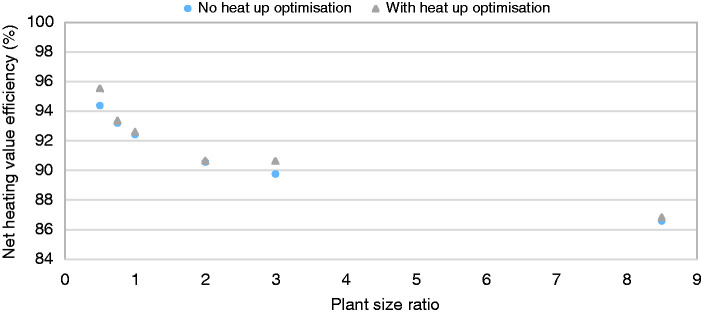
Net heating value boiler efficiency across PSR, with and without heat up optimisation.

Cycling is a clear symptom of oversizing of the heating system with respect to the building heat demand. The defining features of the modern heating systems that contribute to this undesired behaviour are sizing and control. The boiler is unable to modulate sufficiently low to match the required demand for heat due to both the oversizing of the boiler with respect to the building heat demand combined with its finite modulation range, where the minimum output is set as a fixed percentage of maximum output. In the case of combi boilers, this is dependent on peak hot water demand, not space heating. Control of the boiler modulation also plays a role and simple on/off thermostatic control can limit the ability of a boiler to match the space heating requirement in cases where the modulation range allows it. The duration of the heating operation period also plays a role, as discussed above, with a higher PSR required to meet the additional power requirements of warming up the building after enforced intermittent operation from the heating schedule. The impact of PSR on efficiency (accounting for gas and electrical consumption of the appliance) was investigated, see Figure 6, showing that in these simulations the system efficiency decreased as PSR increased, corresponding to an increase in cycling behaviour.

Optimum efficiency of boilers is reached during sustained steady state conditions with low water temperature in the heating circuit. For intermittent heating and variable external temperatures, the interplay of operational parameters becomes more complex and it is generally held that longer operating times can be conducive to improved efficiency due to the lower impact of standby losses and the potential^7^ for lower average flow temperatures coming from the boiler and returning from the radiators. However, despite the longer running times, Figure 6 shows that the net efficiency effect of the ‘heat up optimisation’ function is marginal, with less than 0.5% difference in efficiency for PSR 0.5 and no difference at the largest PSR of 8.5.

## Internal temperature and energy demand

**Figure 7.**
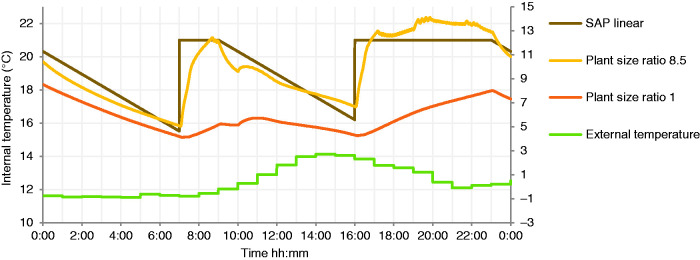
Internal living area and external air temperature for SAP and PSR 8.5, PSR 1 without ‘Heat up optimisation’.

In intermittent operation, it is striking that an oversized boiler system, with PSR of 8.5, is unable to reach the required internal temperature for most of the 2 h long morning heating period during cold weather, as shown in Figure 7. All smaller boilers in the simulations also failed to meet the setpoint temperature in the morning heating period. The PSR 1 boiler, for example, would have been unable to deliver reasonable comfort throughout the colder winter days, also shown in Figure 7.

**Figure 8.**
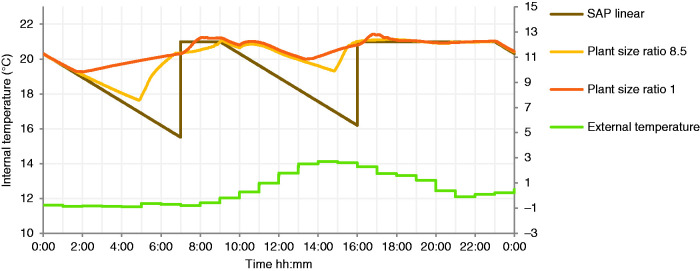
Z1 Internal temperatures across PSR with ‘heat up optimisation’. SAP: standard assessment procedure.

The alternative control strategy, to simple intermittent boiler timing, considered here is the ‘heat up optimisation’ function, which aims to heat the property to achieve setpoint during the specified hours. This can lead to the boiler operating hours earlier than if it only operates at the start of the heating schedule. Heat up optimisation requires an estimate of the thermal properties of the building, and by extending the heating period, it counterbalances the effect of intermittent heating, reducing the need for boiler oversizing, as shown in Figure 8. The longer running hours of systems with heat up optimisation are successful in delivering more consistent internal temperatures, close to setpoint in operating hours, than those using a standard intermittent schedule (Figure 7).

**Figure 9.**
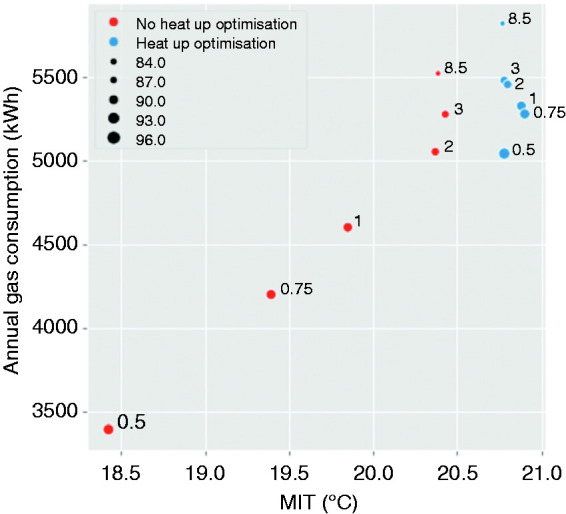
Annual gas consumption plotted against building mean internal temperature (MIT), size of data points indicates efficiency, blue circles for heat up optimisation active, data labels show PSR.

The relationship between energy demand and the mean internal temperature (MIT) of the property, for different PSR, with and without heat up optimisation is shown in Figure 9. Simulations with a fixed heating schedule and no heat up optimisation, shown in red, show a steady increase in gas demand with MIT for PSR 0.5 to 2. This is associated with an increase in boiler output achieving higher temperatures during the schedule; with a corresponding increase in energy demand, the reduction in efficiency (Figure 6) has only a small effect. However, for PSRs greater than 2, the drop in efficiency is clear: there is no significant change in internal temperature despite an increase in gas demand.

Whilst low PSRs of 1, 0.75 and 0.5 delivered the highest efficiencies (Figure 6), when constrained by a fixed heating schedule, with the resultant intermittent heating, they were unable to provide enough heat to raise the room temperature to the desired setpoint. This led to a corresponding drop in MIT and heat energy delivered. However, the ‘heat up optimisation’ function has overcome this issue, leading to a longer effective heating period and delivering higher MIT than the simulations without this feature (Figure 9). Importantly, the results for heat up optimisation control show that the same MIT is provided at an increasing efficiency as the PSR decreases, offsetting the increased operational periods. Thus, the dwelling can achieve higher mean internal temperature with less energy demand by operating longer.

## Discussion

The oversizing of boilers is endemic in the UK stock,^6^ and therefore the associated efficiency reduction caused by on/off cycling is likely to be systemic, causing widespread underperformance of boiler heating systems. The simulations with lower boiler thermal output ranges and PSR better matched heat demand and supply across a year. However, whilst this improved efficiency, it did so at the expense of thermal comfort: the dwelling did not achieve setpoint temperature during the morning heating period for this intermittent schedule. Indeed, even with a PSR of 8.5, the internal temperature did not achieve setpoint for significant parts of the schedule. A key cause of this inability of the heating system to meet the demanded temperatures is the ability to distribute heat throughout the property: it is limited by the pump flow rate, pipework and the size of emitters. Increasing emitter size is likely to improve the system’s ability to deliver the demanded temperature, although the potential impact on cycling, and efficiency, is not clear but should be considered in the context of hydraulic parameters such as pump control, hydraulic balancing and thermostatic radiator valves.

The property simulated in this study has above average thermal performance, in EPC band C, score 80, with a heat loss of 136 W/K compared to an average in the UK of approx. 300 W/K.^27^ The link of PSR to efficiency enables the detrimental efficiency effect of cycling across houses with different heat loss (either through construction or retrofit) and across seasons to be estimated since both can be interpreted as a change in effective PSR. A property with the same number of occupants and bathrooms, but worse levels of insulation, would have the same water heating demand, used to set the boiler size, but higher space heat demand. Such lower insulated properties would, in effect, have a smaller PSR and therefore experience less boiler cycling, and lower associated efficiency reduction; however, they would still experience significant penalty.

The findings of this research have implications for policymaking, and the way policies are manifested through regulations. Real boilers are classified according to their measured efficiency and are required to have an energy label (EuP legislation) displaying the rated efficiency. Measurements are made under steady state conditions in the laboratory with fixed flow and return temperatures at maximum and 30% modulation level according to the current standard.^13^ A weighted combination of these efficiency values is made to display on the energy label or use in national calculation models such as SAP for the creation of EPC. Although such measurements are not meant to be accurate predictions of real-world performance, they should be indicative of the relative benefits of products in the case of EuP labelling, and of system performance, in the case of EPCs. The results here show that the same boiler type can operate at significantly different efficiencies according to its relative size compared to the building heat load. Adjusting the boiler efficiency according to its relation to the expected heat demand, for example from a SAP estimate, would improve the accuracy of boiler energy labels and the resulting building energy calculations.

Incorporating the PSR and modulation range into the SAP methodology, and the resulting EPCs, could be simply undertaken without extra or time-consuming assessment criteria. The make and model of boiler is already collected as part of the assessment of the heating system, supplementing this with the boiler size (both minimum and maximum thermal output) from the boiler dataplate or documentation would facilitate estimation of its ability to match the building heat demand. The efficiency losses associated with a consequent boiler cycling may then be estimated, either using a standard adjustment curve, or ideally by using the testing results for the specific boiler. Similarly, SEDBUK ErP labelling may be modified to support improved performance across wider power ranges, lower than the 30% minimum modulation currently used, to better match heat demand. Such measures may incentivise the installation of lower power boilers, combined with appropriate controls, such as heat up optimisation, as well as incentivise manufacturers to produce heating systems capable of operating at high efficiency across a range of outputs that relate to the real heat losses of dwellings.

Even a moderate increase of boiler stock efficiency can have significant carbon abatement potential, in the UK approximately 3000 GWh of gas is saved per 1% improvement,^28^ equating to 612 MtCO_2_ per annum: a significant low cost saving on the way to net zero carbon. Besides the ‘quick win’ carbon saving potential with the existing stock of boilers, the relevance of correct heating system sizing is even more relevant for low carbon heating technologies. The efficiency of systems such as heat pumps is known to be more sensitive to system specification than boilers; bringing in measures of system performance grounded in real-world operation, and tools to support their optimisation, will support the transition to low carbon heating.

## Conclusions

This paper investigates the potential for reducing the energy required to heat homes with boilers, the dominant heating technology in the UK, by addressing their sizing, control and system specification. Dynamic simulation of the heating system, and the building within which it is located, has been undertaken to investigate causes and potential solutions to system underperformance. In particular, this research focuses on the link between the space heating oversizing of boilers and on/off cycling.

Simulations of the dynamic performance of boiler heating systems within a UK dwelling have shown that typical oversizing (PSR 8.5, 28 kW boiler) resulted in over 50 unnecessary CH cycles per day (corresponding closely with field observations of a median of 53^6^) and efficiency of less than 88%. The modelled efficiency of the PSR 8.5 boiler system is 4% lower than minimum Part L Building Regulation requirements on which carbon budget projections and SAP assessment are made, and is associated with a minimum modulation level that is significantly higher than the heat demand for the house in most external conditions. Reducing this PSR to 1, thereby also lowering the minimum modulation level by a factor of 8.5, was found to improve efficiency by 4%, ∼92% efficiency, with further efficiency improvements associated with lower PSR. Simulations for low PSR boilers may not deliver the required internal temperatures throughout the year, but illustrate the impact of minimum modulation level on the system efficiency. This loss of efficiency, caused by a mismatch between the building heat demand and the minimum modulation level at which the boiler can operate, is associated with repeated on/off cycling, which increases electrical losses associated with necessary start-up/shutdown sequences and reduces condensing, due to the inconsistent return temperature. The link between on/off cycling and efficiency enables the former to be used as a proxy for good system operation, and, as a simple parameter to measure, may be employed to diagnose performance issues in the stock.

Whilst efficiency is an important metric of heating system performance, CO_2_ emissions and energy bills are dependent on total gas consumption: system efficiency must be combined with the duration and power of operation. Additionally, the function of the heating system should be to adequately fulfil the occupant(s) comfort requirement and deliver the expected internal temperatures, regardless of the theoretical efficiency of the system. The heating system should be designed and operated to maintain the minimum required internal temperature for the lowest end energy demand.

The simulated results show that, in the case of the building modelled, a boiler sized closer to a PSR of 1 with heat up optimisation would be able to maintain a mean internal temperature at the level requested, better achieving the morning heating setpoint (Figures 7 and 8), for a lower energy consumption than an oversized boiler with PSR 8.5 and without heat up optimisation. This suggests that installing a boiler with PSR significantly smaller than typical, or with a lower minimum modulation level, but using heat up optimisation, would be preferable in terms of emissions and achieving thermal comfort. Simulation of the impact of decreasing the lowest heat output of boilers on total national carbon emissions is the subject of further research, which could be achieved through widening modulation ranges of appliances or curbing the trend for oversizing based on peak DHW demand. All options to address this issues depend on the thermal properties of the stock, the characteristics of existing boilers, and assumptions of the heating use.

The decrease in energy use associated with lower PSR boilers and heat up optimisation leads to both raised internal temperatures outside the heating schedule and to a decreased capability to deliver on demand hot water. Whilst increased temperatures outside heating schedules may deliver some advantages to occupants, studies of hybrid heat pumps^29^ and heat pumps^30^ have highlighted potential sleep disruption from warmer night time temperatures; mitigation strategies may be required. The rate of hot water delivery from low PSR combi boilers may also be insufficient to meet consumer demands, falling below the sizing guidelines currently employed, requiring the use of water storage, with associated space requirements, to deliver the required flow rate.

Fundamental hardware issues of plant size have lasting implications for the efficiency of the system and simple measures, such as software and changed schedules, will not be sufficient to compensate for a problematic underlying system specification. For example, extending the anti-cycle time (the parameter which defines minimum time between starts) may alleviate the problem slightly but risks inadequate heating control and customer dissatisfaction.

Regulation informs and restricts the development of the technology it governs (such as the step change to condensing boilers in the UK in 2005^31^), a failure to address the real performance of a technology in regulations, as for boiler efficiency labelling, may lead to its optimisation for the laboratory, rather than the home. Disparities between real-world performance and reported efficiencies of heating appliances have been widely reported, and boilers are no exception.^5,32^ Crucially for consideration here, the reported underperformance of up to 10%^3^ has not been linked to a particular root cause and has been taken as a systematic underperformance when integrated into policy instruments such as SAP and EPCs. The results of the simulations presented in this paper highlight the minimum modulation power, set by the plant size ratio, as a major contributing factor to the boiler performance gap, with implications for the policies and practices governing heating systems.

## Notes

a
https://www.boilerguide.co.uk/articles/what-size-boiler-needed

b
https://www.hometree.co.uk/boiler-size-calculator.html

c 6.5kW ±15% and 7.3kW respectively, based on median values from Chambers & Oreszczyn and the CHM.
